# Influence of cell culture parameters on oxygen diffusion and cell distribution in 3D-porous scaffolds for bone tissue engineering: finite element modeling and experimental verification

**DOI:** 10.1007/s11517-026-03572-6

**Published:** 2026-04-11

**Authors:** Alireza Saatchi, Hadi Seddiqi, Ghassem Amoabediny, Marco N. Helder, Behrouz Zandieh-Doulabi, Jenneke Klein-Nulend

**Affiliations:** 1 Department of Oral Cell Biology, Academic Centre for Dentistry Amsterdam (ACTA), University of Amsterdam and Vrije Universiteit Amsterdam, Amsterdam Movement Sciences, Gustav Mahlerlaan 3004, Amsterdam, 1081 LA The Netherlands; 2 School of Chemical Engineering, College of Engineering, University of Tehran, Enqelab Avenue, P.O. Box 11365-4563, Tehran, Iran; 3 Department of Biomedical Engineering, Research Center for New Technologies in Life Science Engineering, University of Tehran, Tehran, Iran; 4 Department of Oral and Maxillofacial Surgery/Oral Pathology, Amsterdam University Medical Centers-location Vumc and Academic Centre for Dentistry Amsterdam (ACTA), Vrije Universiteit Amsterdam, Amsterdam Movement Sciences, De Boelelaan 1117, Amsterdam, 1081 HV The Netherlands

**Keywords:** 3D-porous scaffold, Bone tissue engineering, Cell distribution, Finite element analysis, Oxygen diffusion

## Abstract

Successful bone tissue engineering using 3D-porous scaffolds is hampered by insufficient oxygen diffusion and non-uniform cell distribution, which are determined by cell culture parameters. Here we aimed to investigate the influence of cell culture parameters on oxygen diffusion and cell distribution in 3D-porous scaffolds during 15-days by finite-element (FE) modeling. Osteosarcoma cells were cultured in 3D-porous silk scaffolds under 0.05, 0.12, and 0.2 mol/m^3^ medium-oxygen to validate FE modeling. Oxygen concentration in scaffolds decreased by enhancing initial cell number seeded, medium-oxygen concentration, and maximum specific cell growth rate, while it increased by enhancing cell motility-coefficient and molecular diffusivity of oxygen-in-the-cell-phase. Cell density increased by enhancing medium-oxygen concentration, maximum specific cell growth rate, cell motility-coefficient, and molecular diffusivity of oxygen-in-the-cell-phase, while it decreased by enhancing initial cell seeding number. The FE modeling results of cell proliferation in scaffolds were not significantly (< 8%) different from experimental results, indicating that FE modeling data were in good agreement with experimental results. In conclusion, initial cell number seeded, medium-oxygen concentration, and maximum specific cell growth rate, but not cell motility-coefficient or molecular diffusivity of oxygen-in-the-cell-phase, are crucial for creating densely cell-populated constructs with uniform cell distribution in 3D-porous scaffolds, informing future scaffold-based bone regeneration strategies.

**Graphical Abstract:**

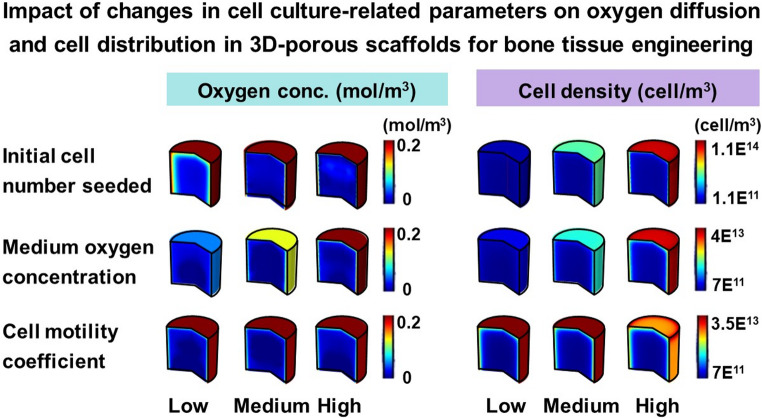

## Introduction

Three-dimensional (3D)-porous scaffolds alleviate the demand arising from the shortage of suitable autograft and allograft for the healing of critical-sized bone defects [1]. They provide the necessary support for cells to proliferate and maintain their differentiated function [2, 3]. 3D-porous scaffolds suffer from limited cell depth viability when cultured in vitro, with viable cells existing within the outer 250–500 μm from the culture medium–scaffold interface [4]. This is mainly due to the lack of oxygen diffusion into the inner regions of the 3D-porous scaffolds [5, 6]. Changes in cell culture-related parameters, i.e. initial cell number seeded [7], and maximum oxygen concentration [8], affect the oxygen diffusion and cell proliferation rate in 3D-porous scaffolds. In addition, cell growth kinetics in the scaffolds determine the cellular behavior and functions in conjunction with oxygen diffusion processes [9]. Discovering optimal cell culture-related parameters is crucial not only to enhance efficacy and functionality of tissue constructs, but also to reduce the cost and time of product preparation [10]. However, optimization of the oxygen diffusion and cell distribution within 3D-porous scaffolds is still a challenge.

Finite element (FE) modeling of 3D-cell cultures helps to better understand the biological and physical factors, e.g., oxygen diffusion, involved in bone tissue formation [11, 12]. Knowledge of the complicated interdependence of 3D-cell cultures is essential for optimizing cell proliferation within 3D-porous scaffolds in vitro [13]. FE modeling can support the search for optimal settings of certain parameters, e.g., oxygen diffusion and cell proliferation [12, 14]. Moreover, FE modeling plays an important role in advancing the field of bone tissue engineering by enabling a better understanding of the relationship between scaffold parameter changes and the final scaffold performance [15, 16].

Static cell culture is a commonly used method in the field of bone tissue engineering, since it mimics the microenvironmental conditions inherent to the human bone cell niche [17, 18]. Static cell cultures inherently limited oxygen diffusion in 3D-porous scaffolds, leading to core hypoxia, which is well-known qualitatively [17, 19, 20]. Optimization of oxygen diffusion as well as cell distribution or density in 3D-porous scaffolds by changing cell culture-related parameters has not been achieved so far. Therefore, in this study we aimed to investigate how changes in cell culture-related parameters affect oxygen diffusion and cell distribution within 3D-porous scaffolds over time by FE modeling. We performed a quantitative parametric analysis of five critical cell culture parameters, i.e., initial cell number seeded, maximum oxygen concentration, maximum specific cell growth, cell motility coefficient, and molecular diffusivity of oxygen in the cell phase, and their influence on oxygen diffusion and cell distribution. A key contribution of our study to the field of bone tissue engineering lies in translating FE modeling predictions into practical design guidelines for achieving more uniform oxygen diffusion and cell distribution in 3D-porous scaffolds under static culture, which is a crucial challenge that prior models have only qualitatively described but rarely quantified for parameter optimization. For this purpose, we first developed a novel FE model, from which we deduced the optimal parameter settings to enhance oxygen diffusion and cell density within the scaffolds. The effect of changes in cell culture-related parameters were put in the context of creating densely cell-populated constructs with uniform spatial cell distribution in a 3D-porous scaffold for bone tissue engineering. We experimentally mimicked and validated the FE modeling results by culturing human osteosarcoma G292 cells in 3D-porous silk scaffolds at different medium oxygen concentrations (0.05, 0.12, and 0.2 mol/m^3^) for 7 days.

## Materials and methods

### FE modeling

We designed an FE model to assess how cell culture-related parameters affect oxygen concentration and cell density within a 3D-porous scaffold using an FE-modeling software package (COMSOL Multiphysics v5.6, Stockholm, Sweden). Three different values of cell culture-related parameters at different levels (high, medium, low) were studied to demonstrate their influence on the distribution of oxygen and cell density in the scaffold.

#### Model geometry

**Fig. 1:**
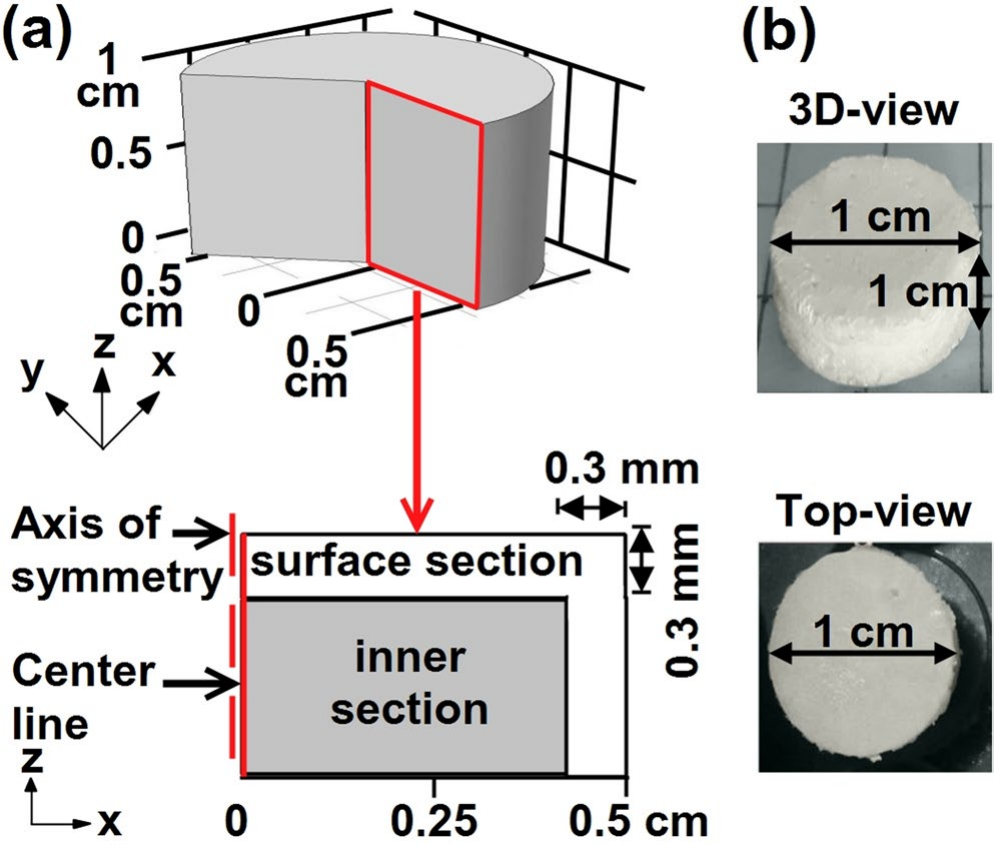
Cylinder-shaped 3D-porous scaffold used in this study. (**a**) 3D and side-view of the scaffold. The surface section of the scaffold was defined as the area located at < 0.3 mm from the scaffold surface in touch with culture medium. The inner section of the 3D-porous scaffold was defined as the area located at > 0.3 mm. The volume percentage of the inner scaffold section was 91% of the total scaffold volume. The center line was used to indicate the oxygen concentration and cell density distribution throughout the depth of the scaffolds. Dashed line: The scaffold’s axis of symmetry. (**b**) 3D and top-view of a 3D-porous scaffold (diameter: 1 cm, height: 1 cm) used to experimentally validate the FE modeling results

A cylinder-shaped scaffold (diameter 1 cm, height 0.1 cm) was constructed in CAD software (COMSOL Multiphysics v5.6, Stockholm, Sweden; Fig. 1a). The inner section of the 3D-porous scaffold was defined as the area located at a distance > 0.3 mm from the surface of the 3D-porous scaffold in touch with culture medium, and the surface section of the scaffold was defined as the area located at a distance < 0.3 mm from the surface of the scaffold in touch with culture medium (Fig. 1a).

#### Model assumptions

The 3D-porous scaffold was assumed to be symmetric, as well as homogeneous, isotropic, and composed of interconnecting pores. The equations were solved in axisymmetric coordinates, enabling spatial gradients along both r and z directions. The mathematical equations were formulated in cylindrical form, and were solved in 2D-axisymmetric cylindrical coordinates, accounting for boundary layers on lateral (r-direction) and top/bottom (z-direction) surfaces. The culture medium was assumed a Newtonian fluid with incompressible properties. Culture medium specifications, i.e. viscosity, temperature, and pH, were assumed constant. The initial cell seeding was assumed to be uniform, and the cells were assumed to stay attached on the scaffolds.

#### Mathematical equations

Mathematical equations were used for 3D-porous scaffolds. Cell growth (Eq. (1)), cell motility (Eq. (2)), growth yield (Eq. (3), oxygen consumption by cells (Eq. (4)), two-phase mass transfer (Eq. (5), and oxygen transfer (Eq. (6)) equations were solved simultaneously by FE modeling to determine the oxygen concentration and cell distribution in a 3D-porous scaffold.

The Monod equation was used as a mathematical model for cell growth (Eq. (1)) [21]:

1 $$\:{\upmu\:}={{\upmu\:}}_{\mathrm{m}\mathrm{a}\mathrm{x}}\frac{\mathrm{C}}{\mathrm{C}+{\mathrm{K}}_{\mathrm{s}}}$$

where $\:{\upmu\:}$ is the cell’s specific growth rate, $\:{{\upmu\:}}_{\mathrm{m}\mathrm{a}\mathrm{x}}\:$is the cell’s maximum specific growth rate, C is the oxygen concentration, and $\:{\mathrm{K}}_{\mathrm{s}}$ is the half velocity constant, which is equal to the concentration of the limiting substrate when the specific growth rate is equal to half of the maximum. To determine cell density, cell motility was considered according to Eq. (2) [9, 22]:

2 $$\:\frac{\partial\:{\uprho\:}}{\partial\:\mathrm{t}}=\frac{1}{\mathrm{r}}\frac{\partial\:}{\partial\:\mathrm{r}}\left({\mathrm{D}}_{\mathrm{c}\mathrm{e}\mathrm{l}\mathrm{l}}\mathrm{r}\frac{\partial\:{\uprho\:}}{\partial\:\mathrm{r}}\right)+{\upmu\:}{\uprho\:}\left(\mathrm{r}.\mathrm{z}.\mathrm{t}\right)$$

where $\:{\uprho\:}\left(\mathrm{r}.\mathrm{z}.\mathrm{t}\right)$ is the cell density in the scaffold, t is time, r is radius, and $\:{\mathrm{D}}_{\mathrm{c}\mathrm{e}\mathrm{l}\mathrm{l}}$ is the cell motility coefficient. The relationship between oxygen consumption by the cells and cell growth is presented in Eq. (3):

3 $$\:{\mathrm{Y}}_{\mathrm{X}\mathrm{S}}=\frac{{{\upmu\:}}_{\mathrm{m}\mathrm{a}\mathrm{x}}}{{\mathrm{V}}_{\mathrm{m}\mathrm{a}\mathrm{x}}-{\mathrm{m}}_{\mathrm{s}}}$$

where $\:{\mathrm{Y}}_{\mathrm{X}\mathrm{S}}$ is the growth yield, which is defined as the amount of oxygen consumption per cell, $\:{\mathrm{V}}_{\mathrm{m}\mathrm{a}\mathrm{x}}$ is the maximum oxygen consumption rate, and $\:{\mathrm{m}}_{\mathrm{s}}$ is the minimum oxygen concentration required to keep the cells alive. The minimum oxygen concentration $\:{\mathrm{m}}_{\mathrm{s}}$ for mammalian cells is very low and can be neglected [11].

Culture medium provides oxygen and nutrition to the cells. The rate of oxygen uptake by the cells is described in Eq. (4):

4 $$\:\mathrm{S}\left(\mathrm{r}.\mathrm{z}.\mathrm{t}\right)=\left(\frac{{{\upmu\:}}_{\mathrm{m}\mathrm{a}\mathrm{x}}}{{\mathrm{Y}}_{\mathrm{X}\mathrm{S}}}+{\mathrm{m}}_{\mathrm{s}}\right){\uprho\:}\left(\mathrm{r}.\mathrm{z}.\mathrm{t}\right)\frac{\mathrm{C}\mathrm{o}(\mathrm{r}.\mathrm{z}.\mathrm{t})}{{\mathrm{K}}_{\mathrm{s}}+\mathrm{C}\mathrm{o}(\mathrm{r}.\mathrm{z}.\mathrm{t})}$$

where $\:\mathrm{S}\left(\mathrm{r}.\mathrm{z}.\mathrm{t}\right)$ is the oxygen uptake rate by cells, z is the scaffold height, and $\:\mathrm{C}\mathrm{o}(\mathrm{r}.\mathrm{z}.\mathrm{t})$ is the dissolved oxygen (DO) concentration in the culture medium. The relationship between the oxygen concentration in fresh medium and the surface layer of cells is presented by the two-phase mass transfer equation in Eq. (5):

5 $$\:{\mathrm{C}}_{\mathrm{I}}={\mathrm{K}}_{\mathrm{e}\mathrm{q}}{\mathrm{C}}_{\mathrm{I}\mathrm{I}}$$

where $\:{\mathrm{C}}_{\mathrm{I}}$ is the oxygen concentration in fresh medium, $\:{\mathrm{K}}_{\mathrm{e}\mathrm{q}}$ is the equilibrium coefficient, and $\:{\mathrm{C}}_{\mathrm{I}\mathrm{I}}$ is the oxygen concentration in the surface layer of cells. The mass transport equation for oxygen is presented in Eqs. (6) and (7) [11]:

6 $$\:\frac{\partial\:\mathrm{C}}{\partial\:\mathrm{t}}+\nabla\:.\mathrm{J}+\mathrm{u}.\nabla\:\mathrm{c}=\mathrm{S}\left(\mathrm{r}.\mathrm{z}.\mathrm{t}\right)$$

7 $$\:J={\mathrm{D}}_{{\mathrm{O}}_{2,\mathrm{e}\mathrm{f}\mathrm{f}}}\nabla\:.\mathrm{c}\:$$

where $\:{\mathrm{D}}_{{\mathrm{O}}_{2,\mathrm{e}\mathrm{f}\mathrm{f}}}$ is the effective oxygen diffusion coefficient and u is the flow velocity. The system was assumed to be static, and thus u was set to zero. Therefore, Eq. (6) was rewritten as Eq. (8):

8 $$\:\frac{\partial\:\mathrm{C}}{\partial\:\mathrm{t}}+\nabla\:.\mathrm{J}+\mathrm{u}.\nabla\:\mathrm{c}=\mathrm{S}\left(\mathrm{r}.\mathrm{z}.\mathrm{t}\right)$$

The effective oxygen diffusion coefficient in porous media was calculated by using Eq. (9) [11]:

9 $$\:\frac{{\mathrm{D}}_{{\mathrm{O}}_{2,\mathrm{e}\mathrm{f}\mathrm{f}}}}{{\mathrm{D}}_{{\mathrm{O}}_{2}}}=\frac{2(1-{{\upepsilon\:}}_{(\mathrm{r}.\mathrm{z}.\mathrm{t})})}{2+{{\upepsilon\:}}_{(\mathrm{r}.\mathrm{z}.\mathrm{t})}}$$

where $\:{\mathrm{D}}_{{\mathrm{O}}_{2}}$ is molecular diffusivity of oxygen in cell phase, and $\:{{\upepsilon\:}}_{(\mathrm{r}.\mathrm{z}.\mathrm{t})}$ is the cell volume fraction. The cell volume fraction in Eq. 9 refers to the macroscopic volume fraction of cells within the homogenized scaffold region. The cell volume fraction $\:{{\upepsilon\:}}_{(\mathrm{r}.\mathrm{z}.\mathrm{t})}$ is described as follows by Eq. (10):

10 $$\:{{\upepsilon\:}}_{\left(\mathrm{r}.\mathrm{z}.\mathrm{t}\right)}={\mathrm{V}}_{\mathrm{c}\mathrm{e}\mathrm{l}\mathrm{l}}\:{\uprho\:}\left(\mathrm{r}.\mathrm{z}.\mathrm{t}\right)$$

where $\:{\mathrm{V}}_{\mathrm{c}\mathrm{e}\mathrm{l}\mathrm{l}}$ is the cell volume.

#### Initial values and boundary conditions

The initial values used for FE modeling were as follows: Initial oxygen concentration throughout the scaffold at day 0 was assumed to be uniform and equal to the medium oxygen concentration at the surface (0.2 mol/m³) as the scaffold is fully equilibrated with the medium prior to culture initiation; Initial porosity (ɛ): 0.8; Volume of a cell (Vcell): 560 × 10^− 18^ m^3^ [23]; Operational temperature (T): 37 °C; Maximum oxygen consumption rate (Vmax): 1.75 × 10^− 17^ mol/(cell.s) [24]; Half velocity constant (Ks): 0.01 × 10^5^ mol/m^3^ [25]; Equilibrium coefficient (Keq): 0.1; Initial cell number seeded (ρinitial): 10 × 11 cells/m^3^ (low; [26]), 10^12^ cells/m^3^ (medium; [27]), and 2 × 10^12^ cells/m^3^ (high). Cell motility coefficient (Dcell): 10^− 11^ cm^2^s^− 1^ (low; [28]), 10^− 10^ cm^2^s^− 1^ (medium; [28]), and 10^− 9^ cm^2^s^− 1^ (high; [28]); Maximum specific cell growth rate (µ_max): 11 × 10^− 6^ s^− 1^ (low; [29]), 5 × 10^− 6^ s^− 1^ (medium; [9]), and 10^− 5^ s^− 1^ (high; [26]); Molecular diffusivity of oxygen in cell phase (DO2): 0.8 × 10^− 9^ m^2^s^− 1^ (low; [29]), 1.6 × 10^− 9^ m^2^s^− 1^ (medium; [26]), and 3.2 × 10^− 9^ m^2^s^− 1^ (high).

The boundary conditions used for FE modeling were as follows: Zero-flux (∂ρcell/∂*n* = 0) boundary condition was applied for cell density. The medium oxygen concentration on the scaffold surface was constant, and equal to the oxygen concentration in fresh medium. To evaluate the effect of oxygen concentration in fresh medium (C_0_) on oxygen diffusion and cell proliferation, the oxygen concentration in fresh medium was assigned as a boundary condition with values of 0.05 mol/m^3^ (low; [30]), 0.12 mol/m^3^ (medium), and 0.2 mol/m^3^ (high; [12]).

#### Mesh generation

To generate FE-meshes, physics-controlled meshes with normal element size (total elements: 336; element type: tetrahedral) were constructed using COMSOL Multiphysics v5.6.

### Experimental method

For verification of the FE modeling data by experimental results using cell culture, 3D-porous silk scaffolds (diameter 1 cm, height 1 cm) were fabricated (Fig. 1b).

#### Silk scaffold fabrication, morphology, and microstructure

Fibroin protein was extracted from silkworm cocoons as described [31]. Briefly, 1.25 g dried cocoons were boiled for 90 min in 500 ml of 0.02 M sodium carbonate solution (Na_2_CO_3_; Merck KGaA, Darmstadt, Germany) to remove sericin. The degummed silk fibers were dried in a fume hood for 48 h. Isolated, dry fibroin was dissolved in 9.3 M lithium bromide solution (Sigma Aldrich, Steinheim, Germany) for 5 h, dialyzed against water using dialysis membrane (MWCO 12000 Da, Sigma Aldrich, Milan, Italy), and centrifuged at 8500 rpm for 30 min. The silk fibroin blends were put in stainless steel molds (diameter 1 cm, height: 1 cm) to prepare 3D-porous scaffolds using a freeze-drying technique. 3D-porous silk scaffolds were cross-linked and sterilized by autoclaving, placed in 6-well culture plates (Greiner, Bio-one, Alphen a/d Rijn, Netherlands), and washed four times with phosphate-buffered saline (PBS) before cell seeding.

### Cell culture

Human osteosarcoma cells G292 (National Cell Bank of Iran, Tehran, Iran) were grown in RPMI-1640 medium (BIO-IDEA, Tehran, Iran) supplemented with 10% fetal bovine serum (FBS; Gibco, Paisly, UK), gentamycin (50 µg/ml; Gibco), and 1% penicillin-streptomycin (Gibco). G292 cells were seeded at a density of 5 × 10^5^ cells/m^3^ scaffold (scaffold diameter 1 cm, height 1 cm). The cell-seeded scaffolds were cultured in medium with an oxygen concentration of 0.2 mol/m^3^ for 1 day in a humidified incubator with 5% CO_2_ in air at 37 °C. For experiments, cells were cultured in medium with an oxygen concentration of 0.05, 0.12, or 0.2 mol/m^3^ at 37 °C in a humidified atmosphere of 5% CO_2_ in air for 7 days. Finally, scaffolds were washed six times with PBS, fixed with 4% paraformaldehyde for 60 min at room temperature, followed by overnight fixation at 4 °C, and washed with deionized water.

### Cell proliferation

Cell proliferation was assessed by determining the cell number in the scaffolds at days 3 and 7, and by dividing these numbers to the cell number in the scaffolds at day 0, using AlamarBlue^®^ fluorescent assay (Invitrogen, Frederick, MD, USA), according to the manufacturer’s instructions [2]. We found a linear relationship between AlamarBlue^®^ fluorescence and cell number (data not shown). At each time point, scaffolds were transferred to 6-well plates containing fresh RPMI-1640 medium with 10% AlamarBlue^®^. Fresh medium was added to each well until it completely covered the scaffolds. Then scaffolds were incubated in AlamarBlue^®^ solution for 4 h in a humidified incubator with 5% CO_2_ in air at 37 °C. The solution was harvested from the scaffolds, which were placed in new wells with fresh medium. The fluorescence intensity of the collected AlamarBlue^®^ solution was measured at 530 nm with a Synergy HT^®^ spectrophotometer. Scaffolds were analyzed in triplicate.

### Statistical analysis

All experiments were carried out in triplicate. Data are presented as mean ± standard error of the mean (SEM). The two-way ANOVA was used to test statistical significance of data on comparison of experimental and FE modeling results. Differences were considered significant if *p* < 0.05. Statistical analysis was performed by GraphPad Prism 9 (GraphPad Software, San Diego, CA, USA).

## Results

A 3D-porous scaffold (diameter: 1 cm, height: 1 cm) was used for FE modeling (Fig. 1a). The surface section of the 3D-porous scaffold was defined as the area located at < 0.3 mm from the surface of the scaffold in touch with culture medium. The inner section of the 3D-porous scaffold was defined as the area located at > 0.3 mm from the surface of the scaffold in touch with culture medium. The volume percentage of the surface section of the 3D-porous scaffold was 8.8% of the total scaffold volume (Fig. 1a). The center line was used to indicate the oxygen concentration and cell density distribution throughout the scaffold, from top to bottom (Fig. 1a). The 3D-view and top view of the 3D-porous silk scaffolds show the dimensions (diameter: 1 cm, height: 1 cm) of the scaffolds used to experimentally validate the FE modeling results (Fig. 1b).

- **FE modeling results**

Changes in cell culture-related parameters after 5, 10, and 15 days affected oxygen and cell percentages within the surface section of the 3D-porous scaffold (Table 1). Increasing the initial cell number seeded from 10^11^ to 2 × 10^12^ cells/m^3^ increased the percentage of oxygen within the surface section by 2.6-fold after 15 days. Raising the medium oxygen concentration from 0.05 to 0.2 mol/m^3^ increased this percentage by 2.7-fold. Enhancing the cell motility coefficient from 10^− 11^ to 10^− 9^ cm^2^s^− 1^ decreased the percentage of oxygen within the surface section by 0.7-fold. Enhancing the maximum specific cell growth rate from 10^− 6^ to 10^− 5^ s^− 1^ increased this percentage by 2.5-fold. Increasing the molecular diffusivity of oxygen in the cell phase from 0.8 × 10^− 9^ to 3.2 × 10^− 9^ m^2^s^− 1^ reduced the percentage of oxygen within the surface section by 0.7-fold after 15 days. Raising the initial cell number seeded from 10^11^ to 2 × 10^12^ cells/m^3^ increased the percentage of cells in the surface section of the scaffold by 2.5-fold after 15 days. Increasing the medium oxygen concentration from 0.05 to 0.2 mol/m^3^ increased this percentage by 1.9-fold. Increasing the cell motility coefficient from 10^− 11^ to 10^− 9^ cm^2^s^− 1^ reduced this percentage by 0.8-fold, whereas raising the maximum specific cell growth rate from 10^− 6^ to 10^− 5^s^− 1^ increased this percentage by 7.8-fold (Table 1). Increasing the molecular diffusivity of oxygen in the cell phase from 0.8 × 10^− 9^ to 3.2 × 10^− 9^ m^2^s^− 1^ reduced the percentage of cells in the surface section by 0.6-fold after 15 days (Table 1).

**Table 1:** FE modeling of the effect of changes in cell culture-related parameters on the percentage of oxygen and cells within an inner section of a 3D-porous scaffold after 5, 10 and 15 days. The surface section of the 3D-porous scaffold was defined as the area located at ≤ 0.3 mm from the scaffold surface in touch with culture medium. The volume percentage of the surface section of the 3D-porous scaffold was 8.8% of the total scaffold volume

Parameter	Value	% O_2_ in surface section of 3D-porous scaffold					% Cells in surface section of 3D-porous scaffold		
		Day 5		Day 10		Day 15	Day 5	Day 10	Day 15
ρ_initial_ (cells/m^3^)	10^11^	12.50		19.51	28.95		9.90	13.26	20.84
	10^12^	34.86		47.75		65.24	16.07	28.56	45.59
	2 × 10^12^	40.22		58.80		74.64	18.53	33.65	52.93
C_0_ (mol/m^3^)	0.05	27.74		24.90		19.88	12.22	17.11	21.28
	0.12	23.51		37.24		47.36	13.71	21.83	34.07
	0.2	25.18		44.83		54.05	14.28	24.64	40.27
D_cell_ (cm^2^s^− 1^)	1 × 10^− 11^	32.11		60.14		64.56	15.38	26.30	42.47
	1 × 10^− 10^	25.11		39.56		57.22	14.28	24.64	40.27
	1 × 10^− 9^	19.28		28.48		45.24	14.04	23.02	34.43
$\:{{\upmu\:}}_{\mathrm{m}\mathrm{a}\mathrm{x}}$(s^− 1^)	1 × 10^− 6^	18.71		20.47		22.16	9.71	10.71	11.83
	5 × 10^− 6^	25.18		44.83		54.05	14.28	24.64	40.27
	1 × 10^− 5^	41.12		87.89		54.77	26.20	65.05	92.27
D_O2_ (m^2^s^− 1^)	0.8 × 10^− 9^	32.11		60.14		64.56	17.54	31.16	50.53
	1.6 × 10^− 9^	25.11		39.56		57.22	14.34	24.35	40.19
	3.2 × 10^− 9^	19.28		28.48		45.24	12.77	20.05	32.62

O_2_, oxygen concentration; ρ_initial_: Initial cell number seeded; C_0_: Medium oxygen concentration; D_cell_: Cell motility coefficient;$\:{{\upmu\:}}_{\mathrm{m}\mathrm{a}\mathrm{x}}$: Maximum specific cell growth rate; D_O2_: Molecular diffusivity of oxygen in cell phase

**Fig. 2:**
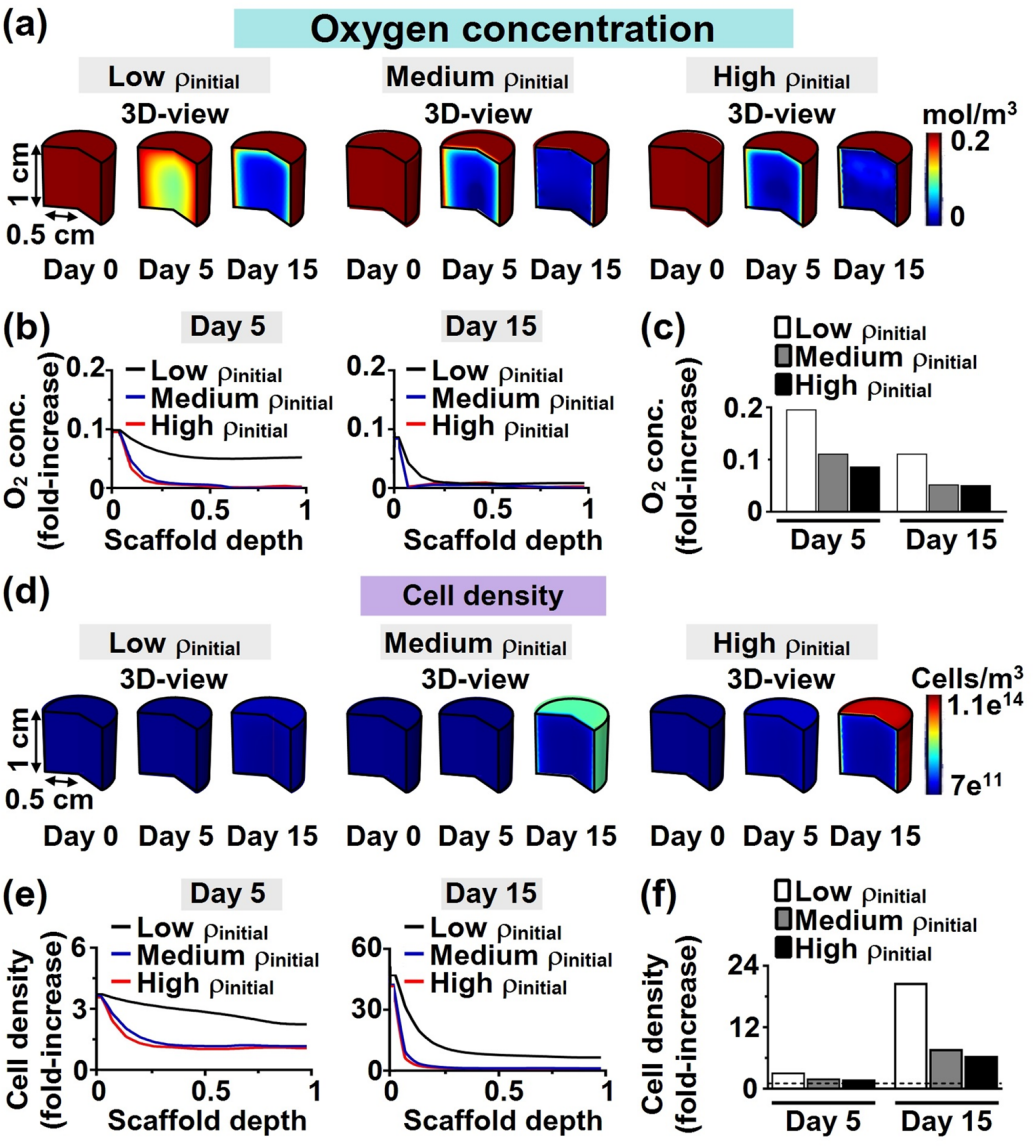
FE modeling of the effect of changes in initial cell seeding number (low: 10^11^, medium: 10^12^, and high: 2 × 10^12^ cells/m^3^) on oxygen concentration and cell density within a 3D-porous scaffold. (**a**) 3D-view of the oxygen concentration magnitude and distribution in the scaffold during 15 days. Initial oxygen concentration throughout the scaffold at day 0 was assumed to be uniform and equal to the medium oxygen concentration at the surface (0.2 mol/m³) as the scaffold is fully equilibrated with the medium prior to culture initiation. (**b**) Oxygen concentration throughout the scaffold, from top to bottom along the center line, after 5 and 15 days. The O₂ concentration-fold increase was defined as the relative change in oxygen concentration compared to the initial medium concentration (C_initial_), calculated as C_t_/C_initial_, where C_t_ is the concentration at time. (**c**) Average oxygen concentration in the scaffold after 5 and 15 days. The oxygen concentration was compared relative to initial value (0.2 mol/m^3^). (**d**) 3D-view of cell density in the scaffold up to 15 days. (**e**) Cell density throughout the scaffold, from top to bottom along the center line, after 5 and 15 days. The cell density was compared relative to Initial cell number seeded (ρ_initial_). (**f**) Average cell density in the scaffold after 5 and 15 days. The cell density-fold increase was defined as the relative change in cell density compared to the initial cell density (C_0_), calculated as C_t_/C_0_, where C_t_ is the concentration at time. ρ_initial_, initial cell number seeded; O_2_ conc., oxygen concentration

The initial cell seeding number (low: 10^11^, medium: 10^12^, and high: 2 × 10^12^ cells/m^3^) affected the magnitude and distribution of oxygen concentration and cell density within 3D-porous scaffolds (Fig. 2). The oxygen concentration in the scaffolds with low, medium, or high cell seeding number was non-uniform and changed over time (Fig. 2a). The oxygen distribution was more homogeneous in scaffolds with high cell seeding number compared to scaffolds with low or medium cell seeding number at all-time points measured. The oxygen diffusion distance in all scaffolds decreased during 15 days of culture. The oxygen concentration near the surface of the scaffolds reached its highest value at day 15, regardless of whether the scaffolds had a low, medium, or high cell seeding number. The oxygen concentration throughout the scaffolds decreased by 0.02–0.08.02.08-fold after 15 days (Fig. 2b). Increasing the initial cell number seeded from 10^11^ to 2 × 10^12^ cells/m^3^ decreased the average oxygen concentration by 0.11-0.05.11.05-fold within the scaffold after 15 days (Fig. 2c). Cell density in the scaffolds with low initial cell seeding number was uniform, but with medium or high initial cell seeding number cell density it was non-uniform and increased over time (Fig. 2d). The cell density in the scaffolds over time showed cell proliferation near the boundary layers in the scaffolds (Fig. 2d). The cell density near the surface of the scaffolds reached to its highest value at day 15, regardless of whether the scaffolds had a low, medium, or high cell seeding number. The cell density throughout the scaffolds decreased by 0.13-0.03-fold after 15 days (Fig. 2e). Increasing the initial cell number seeded from 10^11^ to 2 × 10^12^ cells/m^3^ enhanced the average cell density by 20.70-6.39-fold within the scaffold after 15 days (Fig. 2f).

**Fig. 3:**
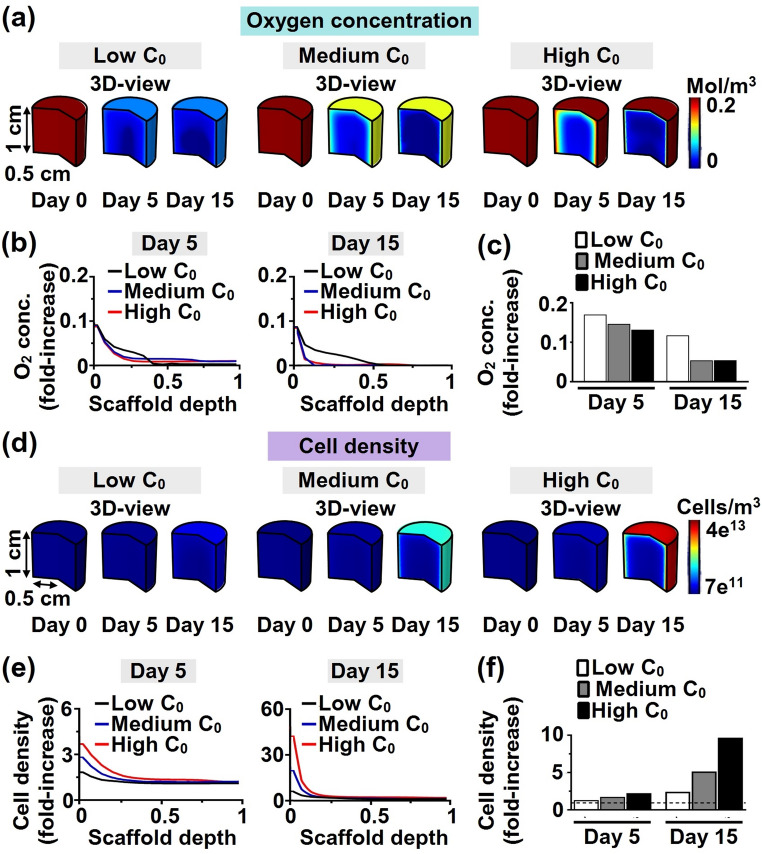
FE modeling of the effect of changes in medium oxygen concentration (low: 0.05, medium: 0.12, and high: 0.2 mol/m^3^) on oxygen concentration and cell density within a 3D-porous scaffold. (**a**) 3D-view of oxygen concentration in the scaffold over time. Initial oxygen concentration throughout the scaffold at day 0 was assumed to be uniform and equal to the medium oxygen concentration at the surface (0.2 mol/m³) as the scaffold is fully equilibrated with the medium prior to culture initiation. (**b**) Oxygen concentration throughout the scaffold, from top to bottom along the center line, after 5 and 15 days. The O₂ concentration-fold increase was defined as the relative change in oxygen concentration compared to the initial medium concentration (C_initial_ = 0.2 mol/m^3^), calculated as C_t_/C_initial_, where C_t_ is the concentration at time. (**c**) Average oxygen concentration in the scaffold after 5 and 15 days. The oxygen concentration was compared relative to initial value (0.2 mol/m^3^). (**d**) 3D-view of cell density in the scaffold over time. (**e**) Cell density throughout the scaffold, from top to bottom along the center line, after 5 and 15 days. The cell density-fold increase was defined as the relative change in cell density compared to the initial cell density (C_0_), calculated as C_t_/C_0_, where C_t_ is the concentration at time. (**f**) Average cell density in the scaffold after 5 and 15 days. The cell density was compared relative to Initial cell number seeded (ρ_initial_). C_0_, Medium oxygen concentration; O_2_ conc., oxygen concentration

The medium oxygen concentration (low: 0.05, medium: 0.12, and high: 0.2 mol/m^3^) changed the magnitude and distribution of oxygen concentration and cell density within 3D-porous scaffolds (Fig. 3). The oxygen concentration in the scaffolds with low, medium, or high medium oxygen concentration was non-uniform and changed over time (Fig. 3a). The oxygen distribution was more homogeneous in the scaffolds with low oxygen concentration in the medium compared to scaffolds with medium or high oxygen concentration at all-time points measured (Fig. 3a). The oxygen diffusion distance in all scaffolds decreased during 15 days of culture (Fig. 3a). The oxygen concentration near the surface of the scaffolds with low, medium, or high medium oxygen concentration reached its highest value at day 15. The oxygen concentration throughout the scaffolds decreased by 0.01-fold after 15 days (Fig. 3b). Increasing the medium oxygen concentration from 0.05 to 0.2 mol/m^3^ decreased the average oxygen concentration by 0.12-0.05-fold within the scaffold after 15 days (Fig. 3c). Cell density in the scaffolds with low oxygen concentration in the medium was uniform, but in the scaffolds with medium and high oxygen concentration the cell density was non-uniform and changed over time (Fig. 3d). The cell density in the scaffolds over time showed cell proliferation near the boundary layers of the scaffolds (Fig. 3d). The cell density near the surface of the scaffolds reached its highest value after 15 days, regardless of whether the medium had a low, medium, or high cell oxygen concentration. The cell density throughout the scaffolds decreased by 0.17-0.04-fold after 15 days (Fig. 3e). Increasing the medium oxygen concentration from 0.05 to 0.2 mol/m^3^ enhanced average cell density by 2.42–9.68-fold within the scaffold (Fig. 3f).

**Fig. 4:**
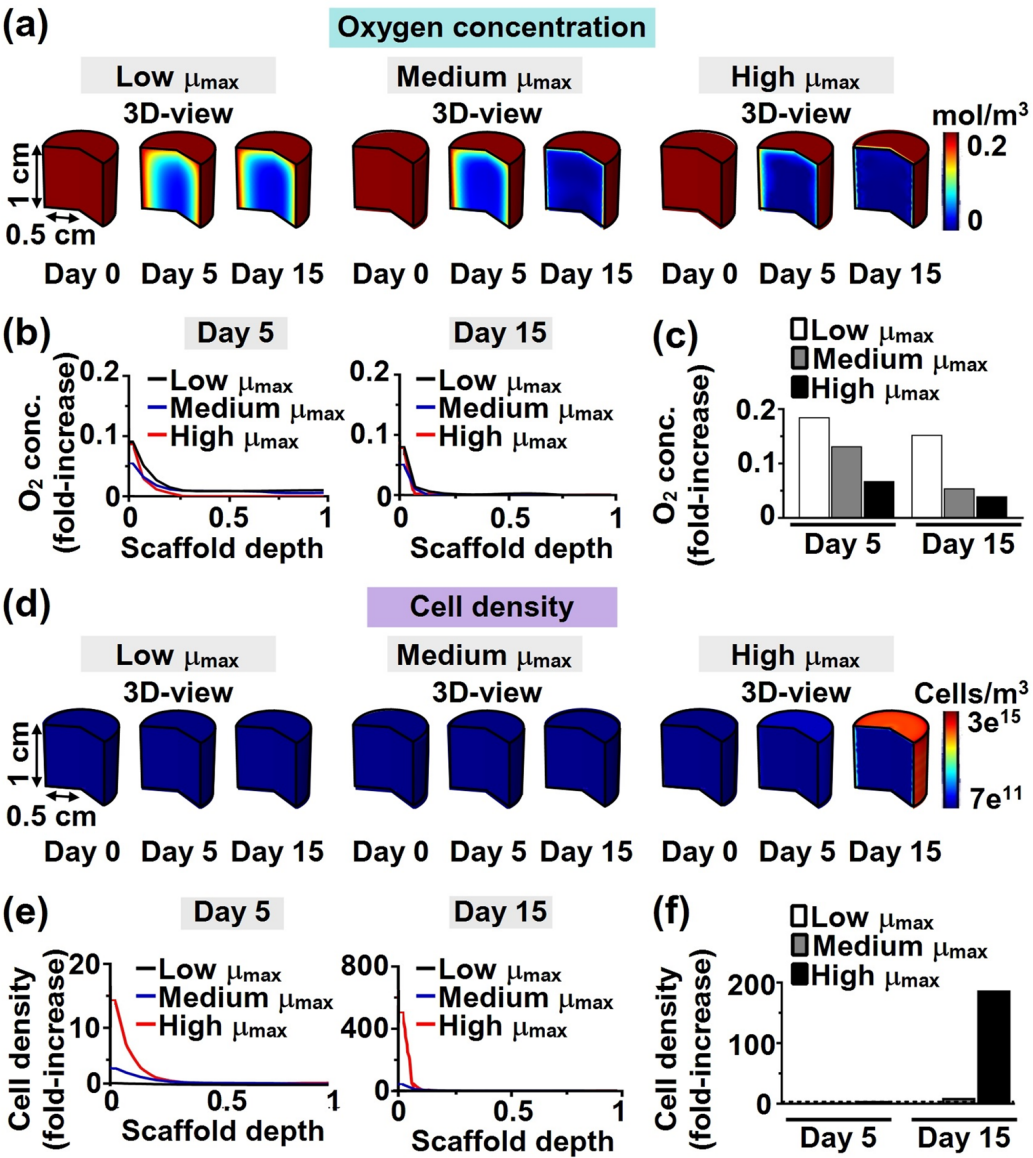
FE modeling of the effect of changes in maximum specific cell growth rate (low: 1 × 10^− 6^, medium: 5 × 10^− 6^, and high: 1 × 10^− 5^ s^− 1^) on oxygen concentration and cell density within a 3D-porous scaffold. (**a**) 3D-view of oxygen concentration in the scaffold over time. Initial oxygen concentration throughout the scaffold at day 0 was assumed to be uniform and equal to the medium oxygen concentration at the surface (0.2 mol/m³) as the scaffold is fully equilibrated with the medium prior to culture initiation. (**b**) Oxygen concentration throughout the scaffold, from top to bottom along the center line, after 5 and 15 days. The O₂ concentration-fold increase was defined as the relative change in oxygen concentration compared to the initial medium concentration (C_initial_ = 0.2 mol/m^3^), calculated as C_t_/C_initial_, where C_t_ is the concentration at time. (**c**) Average oxygen concentration in the scaffold after 5 and 15 days. The oxygen concentration was compared relative to initial value (0.2 mol/m^3^). (**d**) 3D-view of cell density in the scaffold over time. (**e**) Cell density throughout the scaffold, from top to bottom along the center line, after 5 and 15 days. The cell density-fold increase was defined as the relative change in cell density compared to the initial cell density (C_0_), calculated as C_t_/C_0_, where C_t_ is the concentration at time. (**f**) Average cell density in the scaffold after 5 and 15 days. The cell density was compared relative to Initial cell number seeded (ρ_initial_). $\:{{\upmu\:}}_{\mathrm{m}\mathrm{a}\mathrm{x}}$, Maximum specific cell growth rate; O_2_ conc., oxygen concentration

The maximum specific cell growth rate (low: 1 × 10^− 6^, medium: 5 × 10^− 6^, and high: 1 × 10^− 5^ s^− 1^) modulated the magnitude and distribution of oxygen concentration and cell density within 3D-porous scaffolds (Fig. 4). The oxygen concentration in scaffolds with low, medium, or high maximum specific cell growth rate was non-uniform and changed over time (Fig. 4a). Oxygen distribution was more homogeneous in the scaffolds with high maximum specific cell growth rate compared to scaffolds with low or medium maximum specific cell growth rate at all time points measured (Fig. 4a). The oxygen diffusion distance in all scaffolds decreased during 15 days of culture (Fig. 4a). The oxygen concentration near the surface of the scaffolds with low, medium, or high maximum specific cell growth rate reached its highest value after 15 days, regardless of whether the maximum specific cell growth rate was low, medium, or high. The oxygen concentration throughout the scaffolds decreased by 0.01-fold after 15 days (Fig. 4b). Increasing the maximum specific cell growth rate from 1 × 10^− 6^ to 1 × 10^− 5^ s^− 1^ decreased the average oxygen concentration within the scaffold by 0.15-0.04-fold after 15 days (Fig. 4c). Cell density in 3D-porous scaffolds with low (1 × 10^− 6^ s^− 1^) or medium (5 × 10^− 6^ s^− 1^) maximum specific cell growth rate was uniform, but in the scaffolds with high (1 × 10^− 5^ s^− 1^) maximum specific cell growth rate the cell density was non-uniform and changed over time (Fig. 4d). The cell density in the scaffolds over time showed cell proliferation near the boundary layers in the scaffolds (Fig. 4d). The cell density near the surface of the scaffolds reached its highest value at day 15, regardless of whether the maximum specific cell growth rate was low, medium, or high. The cell density throughout the scaffolds decreased by 0.54-0.01-fold after 15 days (Fig. 4e). Increasing the maximum specific cell growth rate from 1 × 10^− 6^ to 1 × 10^− 5^ s^− 1^ enhanced the average cell density by 2.42–9.68-fold within the scaffold (Fig. 4f).

**Fig. 5:**
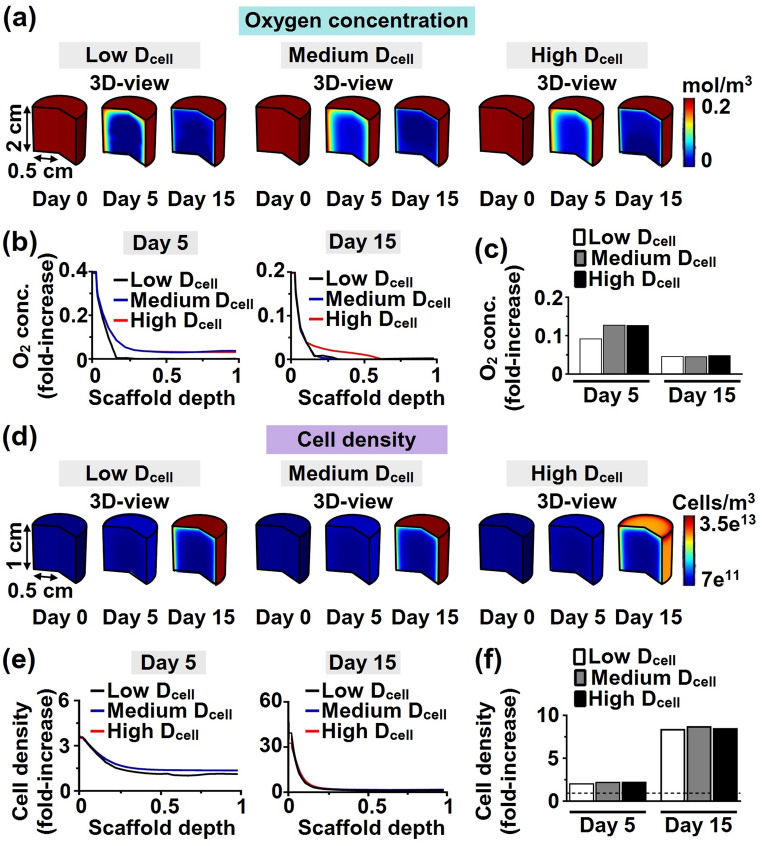
FE modeling of the effect of changes in cell motility coefficient (low: 1 × 10^− 11^, medium: 1 × 10^− 10^, and high: 1 × 10^− 9^ cm^2^s^− 1^) on oxygen concentration and cell density within a 3D-porous scaffold. (**a**) 3D-view 8of oxygen concentration in the scaffold over time. Initial oxygen concentration throughout the scaffold at day 0 was assumed to be uniform and equal to the medium oxygen concentration at the surface (0.2 mol/m³) as the scaffold is fully equilibrated with the medium prior to culture initiation. (**b**) Oxygen concentration throughout the scaffold, from top to bottom along the center line, after 5 and 15 days. The O₂ concentration-fold increase was defined as the relative change in oxygen concentration compared to the initial medium concentration (C_initial_ = 0.2 mol/m^3^), calculated as C_t_/C_initial_, where C_t_ is the concentration at time. (**c**) Average oxygen concentration in the scaffold after 5 and 15 days. The oxygen concentration was compared relative to initial value (0.2 mol/m^3^). (**d**) 3D-view of cell density in the scaffold over time. (**e**) Cell density throughout the scaffold, from top to bottom along the center line, after 5 and 15 days. The cell density-fold increase was defined as the relative change in cell density compared to the initial cell density (C_0_), calculated as C_t_/C_0_, where C_t_ is the concentration at time. (**f**) Average cell density in the scaffold after 5 and 15 days. The cell density was compared relative to Initial cell number seeded (ρ_initial_). D_cell_, Cell motility coefficient; O_2_ conc., oxygen concentration

The cell motility coefficient (low: 1 × 10^− 11^, medium: 1 × 10^− 10^, and high: 1 × 10^− 9^ cm^2^s^− 1^) affected the magnitude and distribution of oxygen concentration and cell density within 3D-porous scaffolds (Fig. 5). The oxygen concentration in the scaffolds with low, medium, or high cell motility coefficient was non-uniform and changed over time (Fig. 5a). The oxygen diffusion distance in all scaffolds decreased during 15 days of culture (Fig. 5a). The oxygen concentration near the surface of the scaffolds reached its highest value at day 15, regardless of whether the cell motility coefficient was low, medium, or high. The oxygen concentration throughout the scaffolds decreased by 0.01-fold after 15 days (Fig. 5b). Increasing the cell motility coefficient from 1 × 10^− 11^ to 1 × 10^− 9^ cm^2^s^− 1^ decreased the average oxygen concentration within the scaffold by 0.05-fold after 15 days (Fig. 5c). Cell density in the scaffolds with low, medium, or high cell motility coefficient was non-uniform and changed over time (Fig. 5d). The cell density in the scaffolds over time showed cell proliferation near the boundary layers in the scaffolds (Fig. 5d). The cell density near the surface of the scaffolds reached its highest value at day 15, regardless of whether the cell motility coefficient was low, medium, or high. The cell density throughout the scaffolds decreased by 0.03–0.05-fold after 15 days (Fig. 5e). Increasing the cell motility coefficient from 1 × 10^− 11^ to 1 × 10^− 9^ cm^2^s^− 1^ enhanced the average cell density by 8.43–8.76-fold within the scaffold after 15 days (Fig. 5f).

**Fig. 6:**
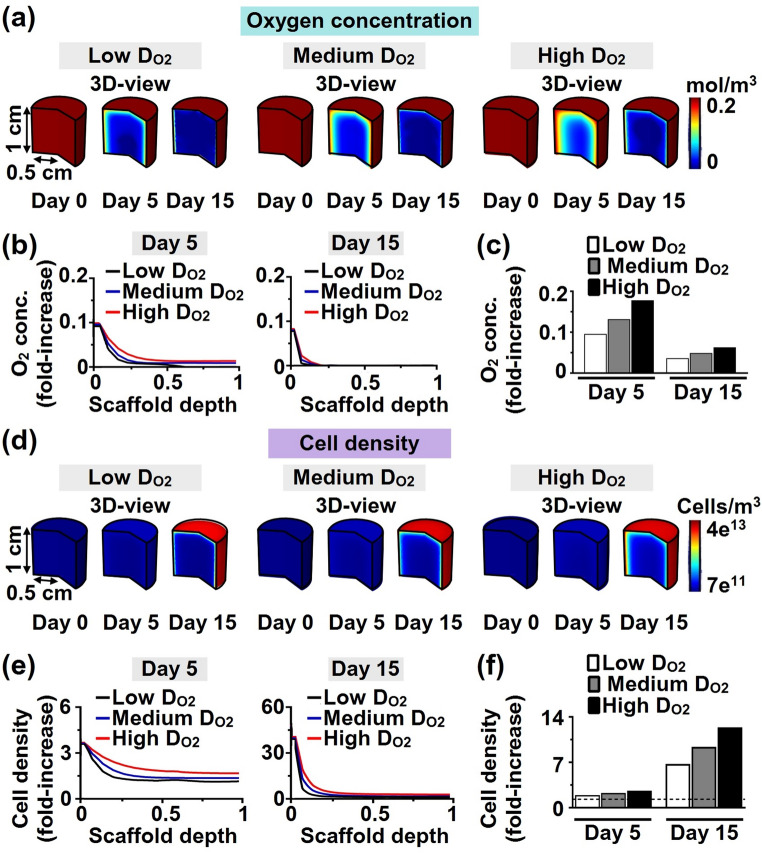
FE modeling of the effect of changes in molecular diffusivity of oxygen in the cell phase (low: 0.8 × 10^− 9^, medium: 1.6 × 10^− 9^, and high 3.2 × 10^− 9^ m^2^s^− 1^) on oxygen concentration and cell density within a 3D-porous scaffold. (**a**) 3D-view of oxygen concentration in the scaffolds over time. Initial oxygen concentration throughout the scaffold at day 0 was assumed to be uniform and equal to the medium oxygen concentration at the surface (0.2 mol/m³) as the scaffold is fully equilibrated with the medium prior to culture initiation. (**b**) Oxygen concentration throughout the scaffold, from top to bottom along the center line, after 5 and 15 days. The O₂ concentration-fold increase was defined as the relative change in oxygen concentration compared to the initial medium concentration (C_initial_ = 0.2 mol/m^3^), calculated as C_t_/C_initial_, where C_t_ is the concentration at time. (**c**) Average oxygen concentration in the scaffold after 5 and 15 days. The oxygen concentration was compared relative to initial value (0.2 mol/m^3^). (**d**) 3D-view of cell density in the scaffold over time. (**e**) Cell density throughout the scaffold, from top to bottom along the center line, after 5 and 15 days. The cell density-fold increase was defined as the relative change in cell density compared to the initial cell density (C_0_), calculated as C_t_/C_0_, where C_t_ is the concentration at time. (**f**) Average cell density in the scaffold after 5 and 15 days. The cell density was compared relative to Initial cell number seeded (ρ_initial_). D_O2_, Molecular diffusivity of oxygen in cell phase; O_2_ conc., oxygen concentration

The molecular diffusivity of oxygen in the cell phase (low: 0.8 × 10^− 9^, medium: 1.6 × 10^− 9^, and high: 3.2 × 10^− 9^ m^2^s^− 1^) affected the magnitude and distribution of oxygen concentration and cell density within 3D-porous scaffolds (Fig. 6). The oxygen concentration in the scaffolds with low, medium, or high molecular diffusivity of oxygen in the cell phase was non-uniform and changed over time (Fig. 6a). The oxygen diffusion distance in all scaffolds decreased during 15 days of culture (Fig. 6a). The oxygen concentration near the surface of the scaffolds reached to its highest value at day 15, regardless of whether the molecular diffusivity of oxygen in the cell phase was low, medium, or high. The oxygen concentration throughout the scaffolds decreased by 6.76–12.40.76.40-fold after 15 days (Fig. 6b). Increasing the cell motility coefficient from 0.8 × 10^− 9^ to 3.2 × 10^− 9^ m^2^s^− 1^ decreased the average oxygen concentration by 0.03-fold within the scaffold (Fig. 6c). Cell density in the scaffolds with low, medium, or high molecular diffusivity of oxygen in the cell phase was non-uniform and changed over time (Fig. 6d). The cell density in the scaffolds over time showed cell proliferation near the boundary layers in the scaffolds (Fig. 6d). The cell density near the surface of the scaffolds reached its highest value at day 15, regardless of whether the molecular diffusivity of oxygen in the cell phase was low, medium, or high. The cell density throughout the scaffolds decreased by 0.01-fold after 15 days (Fig. 6e). Increasing the molecular diffusivity of oxygen in the cell phase from 0.8 × 10^− 9^ to 3.2 × 10^− 9^ m^2^s^− 1^ enhanced the average cell density by 6.76–12.40-fold within the scaffold after 15 days (Fig. 6f).

- **Experimental validation**

**Fig. 7:**
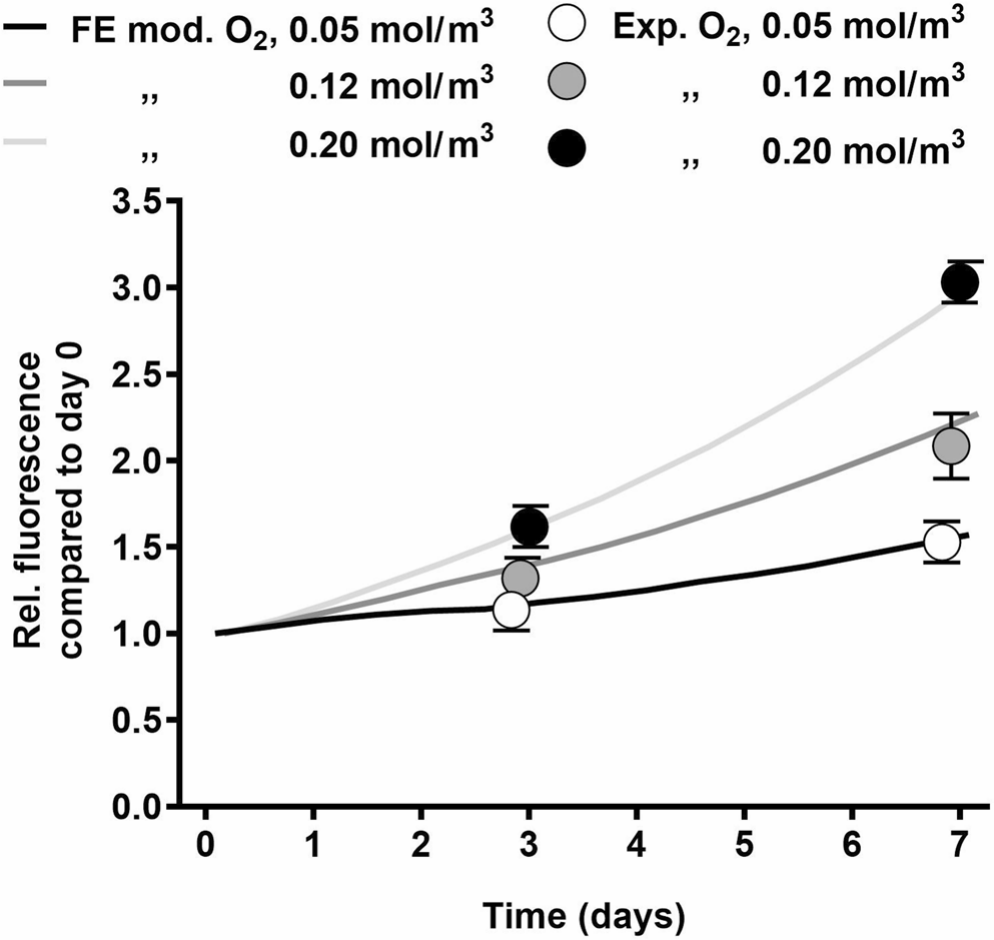
Comparison between experimental and FE modeling results of cell proliferation in 3D-porous silk scaffolds cultured under different medium oxygen concentrations (0.05, 0.12 and 0.2 mol/m^3^) during 7 days. Cell proliferation in the scaffolds was measured after 3 and 7 days and compared with FE modeling results during 7 days. The cell proliferation on the scaffolds was compared relative to day 0. The FE modeling results are in good agreement with experimental results at all-time points measured. FE modeling, finite element modeling; FE mod, Finite element modeling; Exp, experiment

Cell proliferation in 3D-porous silk scaffolds under different medium oxygen concentrations (0.05, 0.12 and 0.2 mol/m3) was assessed experimentally and compared with FE modeling results during 7 days of culture (Fig. 7). Statistical analysis was performed at equivalent time points (days 3 and 7) for both experimental and FE modeling results (Fig. 7). The statistical analysis indicated that the FE modeling results of cell proliferation in the scaffolds were not significantly (maximally 8% at day 7, *p* > 0.05) different from the experimental results (Fig. 7). These findings demonstrate that the FE modeling framework provided in this study was in good agreement with the experimental results and therefore could be validated by the experimental results (Fig. 7). Althought the experimental measurements were limited to the first 7 days of cell culture due to experimental constraints associated with oxygen measurements in the core of the 3D-porous silk scaffolds after 7 days of static culture, the FE-modeling extended to day 15 to predict long-term effects of cell culture parameters on oxygen diffusion and cell distribution. In this study, human osteosarcoma G292 cells were used to validate the FE modeling results, as G292 cells exhibit osteoblast-like characteristics and are widely employed as a representative model for osteoblastic behavior in bone-related studies [32, 33]. Future studies should include pre-osteoblasts or mesenchymal stem cells to enhance the physiological relevance.

## Discussion

The creation of densely cell-populated 3D-porous scaffolds with uniform cell distribution and sufficient oxygen diffusion is a major challenge and limiting factor for bone tissue engineering [34, 35]. We hypothesized that cell culture-related parameters steer oxygen diffusion, oxygen consumption, and cell proliferation within a 3D-porous scaffold over time. However, it is not clear which cell culture-related parameters are most effective to promote oxygen diffusion and cell distribution. Therefore, in the current study, for the first time, we compared the influence of cell culture-related parameters on oxygen diffusion and cell distribution within a 3D-porous scaffold by using FE modeling. We found that: (1) increasing the initial cell number seeded, the medium-oxygen concentration, or the maximum specific cell growth rate, decreased the oxygen diffusion in the scaffold, while increasing the cell motility coefficient or the molecular diffusivity of oxygen in cell phase had the opposite effect; (2) increasing the medium-oxygen concentration, the maximum specific cell growth rate, the cell motility coefficient, or the molecular diffusivity of oxygen in the cell phase, increased the cell distribution within the scaffold over 15 days.

The cell distribution and density within a 3D-porous scaffold are crucial parameters for cell culture and tissue formation [36, 37]. Without enhanced oxygen and nutrient transport, active cells located deeper than ~ 100 μm in a scaffold do not receive sufficient nutrients and oxygen [21]. Cells cultured in 3D-porous scaffolds only survive when they are within ~ 200 μm from the nearest scaffold surface [21]. Therefore, in this study 300 μm was chosen as a boundary between the surface and inner sections of a 3D-porous scaffold based on the maximum distance from the surface of the 3D-porous scaffold. We showed that by increasing the initial cell number seeded, the final cell density in the scaffold also increased after 15 days, but with a marked difference in cell density between the inner and outer parts of the scaffold, i.e. low cell density in the inner parts and high cell density in the outer parts. When a lower initial cell number was seeded, the cell distribution was more uniform. Importantly, there is a maximum value for cell density in a 3D-porous scaffold, and the cell proliferation rate will decrease when the cell density approaches this value. This point will be reached earlier with a higher initial cell seeding density, and consequently the difference in final cell density will be reduced due to a “catch-up” phenomenon from a lower initial cell seeding density [37]. Nevertheless, our results indicate that the cell density in a 3D-porous scaffold enhances by increasing the initial cell number seeded, reaching a maximum by using a maximum initial cell number seeded (2 × 10^12^ cells/m^3^). Our results also indicate that the time needed to increase the cell number to a specified required number can be reduced by increasing the initial cell number seeded.

Oxygen concentration and diffusion are important parameters for cell culture and tissue formation [38]. In 2D-cell culture, medium oxygen concentration will be uniform, and cell density will be homogeneous. However, in a 3D-porous scaffold, the cell distribution directly after cell seeding may be uniform, but this could change dramatically over time during culture. We and others have shown that this may be due to the rapidly increasing cell density in the surface section compared to the inner section of a 3D-porous scaffold over time, with a concomitant reduction in oxygen concentration in the inner section of the scaffold [21, 34]. Efficient cell seeding in 3D-porous scaffolds has been shown to improve bone formation, and thus potentially contributes to successful clinical bone tissue engineering [36]. Lowering the initial cell seeding density would delay the formation of mineralized tissue [39], which is critical to the success of bone tissue engineering using 3D-porous scaffolds, as the premise relies on newly engineered tissue replacing the scaffold over time.

Our results confirm that control of medium oxygen concentration and diffusion within a 3D-porous scaffold is critical to ensure and maintain an optimal cell distribution within the scaffold. One means to optimize cell distribution may be to increase the medium oxygen concentration, as our results indicate that raising the medium oxygen level increases cell density in a 3D-porous scaffold in time. Alternatively, the CO_2_ concentration may be varied, since the ratio between oxygen and CO_2_ is known to affect cell growth and differentiation [40]. The amount of CO_2_ in a CO_2_ tissue culture incubator adjusts the medium oxygen concentration, i.e. with increasing CO_2_, the medium oxygen concentration decreases. Theoretically this would imply that the maximum medium oxygen concentration (0.2 mol/m^3^) and the minimum CO_2_ concentration in a CO_2_ incubator would be optimal to enhance the cell density in 3D-porous scaffolds, but then CO_2_-independent medium should be used to avoid a pH drop.

Increasing the maximum specific cell growth rate resulted in reduced oxygen concentration and higher cell density in a 3D-porous scaffold over time. Oxygen, pH, glucose, osmolality, viscosity, and substance stiffness have been shown to affect cell growth and maximum specific cell growth rate [41, 42]. Increasing the cell density with a higher maximum specific cell growth rate will lower the oxygen concentration in the inner section of the scaffold and raise cell density in the surface part of the scaffold. In contrast, a scaffold with a lower maximum specific cell growth rate will have a more uniform cell distribution. We found that with a maximum specific cell growth rate of 10^− 5^ s^− 1^, the surface section was fully covered by cells before day 15. Hence, our results suggest that the maximum specific cell growth rate should be controlled to prevent decreased penetration depth of living cells into the scaffold.

The cell motility coefficient indicates the ability of cells to move spontaneously within a 3D-scaffold [28]. This implies that for a scaffold with a lower cell motility coefficient, the cell density in the surface section will be higher than in a scaffold with a high cell motility coefficient value, but this may level out upon longer culture periods. We showed that by increasing the cell motility coefficient, the uniformity of spatial distribution of the cells in a 3D-porous scaffold enhanced, but without changing the oxygen concentration or cell density after 15 days of culture.

Increasing the cellular matrix mineralization reduces the molecular diffusivity of oxygen in cell phase. Cellular matrix mineralization during the first week of cell culture in 3D-porous scaffolds hardly occurs. Increasing the cell density would raise cellular matrix mineralization in a 3D-porous scaffold during the second and third week of culture. Therefore, the molecular diffusivity of oxygen in cell phase in a 3D-scaffold becomes higher over time. Increasing the molecular diffusivity of oxygen in cell phase will enhance the distribution of oxygen in the 3D-scaffold, causing a higher cell density in the inner section of the scaffold in time. Thus, increased cell density and molecular diffusivity of oxygen in cell phase will concomitantly positively influence the oxygen distribution in a 3D-porous scaffold.

A uniform initial seeding assumption was applied in the FE modeling to evaluate the effect of cell culture parameters on oxygen diffusion and cell distribtution. Although this assumption may affect the early-phase oxygen distribution and initial cell distribution, the oxygen diffusion gradient in static culture systems rapidly becomes the main factor governing cell proliferation and distribution, particularly during long-term culture. Therefore, the conclusions of our study regarding the influence of cell culture parameters on oxygen diffusion and cell distribution in 3D-porous scaffolds remain valid.

Our FE-modeling, using cell motility and proliferation equations, resulted in cell density distribution over time despite initial uniform seeding. Our FE modeling also resulted in cell accumulation near scaffold boundaries arising from the zero-flux boundary condition applied to cell density. This resulted in restriction of cell transport across scaffold boundaries, but it did not prevent local cell accumulation near the scaffold–medium interface due to oxygen-driven proliferation distribution.

Although the fundamental equations used in this study have been established earlier by others [9, 11, 17, 18], our study provides a quantitative parametric analysis of five key cell culture parameters and their influence on oxygen diffusion and cell distribution in 3D-porous scaffolds. The novelty of the current study lies in the integration of biologically relevant culture parameters into an FE modeling framework, followed by a quantitative parametric analysis across ranges relevant to bone tissue engineering, and in vitro validation of the FE modeling. This analysis had not been performed previously in a unified modeling–experimental framework. Our findings provide practical insights for optimizing 3D-porous scaffold design and fabrication to mitigate hypoxia in the core of these scaffolds, which is a critical barrier to clinical translation.

## Conclusions

In this study, the influence of cell culture-related parameters on oxygen diffusion and cell distribution within a 3D-porous scaffold was studied for the first time using FE modeling and experiments. Our findings provide a quantitative insight into the finetuning of cell culture-related parameters to enhance oxygen diffusion and cell distribution in the scaffold. Our results confirm the hypothesis that cell culture-related parameters steer oxygen diffusion and cell distribution within 3D-porous scaffolds over time. Based on our findings, we suggest that the initial cell number seeded, the medium-oxygen concentration, and the maximum specific cell growth are crucial for creating densely cell-populated constructs with uniform spatial distribution in 3D-porous scaffolds. This may have important implications for bone tissue engineering strategies using cells and 3D-porous scaffolds.

## Data Availability

Dataset available on request from the corresponding author.
